# Rab27b contributes to radioresistance and exerts a paracrine effect via epiregulin in glioblastoma

**DOI:** 10.1093/noajnl/vdaa091

**Published:** 2020-08-08

**Authors:** Soichiro Nishioka, Ping-Hsiu Wu, Toshiaki Yakabe, Amato J Giaccia, Quynh-Thu Le, Hidefumi Aoyama, Shinichi Shimizu, Hiroki Shirato, Yasuhito Onodera, Jin-Min Nam

**Affiliations:** 1 Molecular and Cellular Dynamics Research, Graduate School of Biomedical Science and Engineering, Hokkaido University, Sapporo, Japan; 2 Global Center for Biomedical Science and Engineering, Faculty of Medicine, Hokkaido University, Sapporo, Japan; 3 School of Medicine, Hokkaido University, Sapporo, Japan; 4 Department of Radiation Oncology, Stanford University School of Medicine, Stanford, California, USA; 5 Department of Radiation Oncology, Faculty of Medicine, Hokkaido University, Sapporo, Japan; 6 Department of Radiation Medical Science and Engineering, Faculty of Medicine, Hokkaido University, Sapporo, Japan; 7 Department of Molecular Biology, Faculty of Medicine, Hokkaido University, Sapporo, Japan

**Keywords:** epiregulin, glioblastoma, paracrine, Rab27b, radioresistance

## Abstract

**Background:**

Radiotherapy is the standard treatment for glioblastoma (GBM). However, radioresistance of GBM cells leads to recurrence and poor patient prognosis. Recent studies suggest that secretion factors have important roles in radioresistance of tumor cells. This study aims to determine whether Rab27b, a small GTPase involved in secretory vesicle trafficking, plays a role in radioresistance of GBM.

**Methods:**

Microarray analysis, cell viability analysis, apoptosis assay, immunostaining, and in vivo experiments were performed to assess the effect of Rab27b on radioresistance of GBM. We further investigated paracrine effects mediated by Rab27b after X-ray irradiation using coculture systems of glioma cell lines.

**Results:**

Rab27b was specifically upregulated in irradiated U87MG cells. Furthermore, Rab27b knockdown decreased the proliferation of GBM cells after irradiation. Knockdown of Rab27b in U87MG cells combined with radiation treatment suppressed orthotopic tumor growth in the mouse brain and prolonged the survival of recipient mice. Interestingly, the co-upregulation of Rab27b and epiregulin (EREG), a member of the epidermal growth factor (EGF) family, correlated with radioresistance in glioma cell lines. Additionally, EREG, which was secreted from U87MG cells via Rab27b-mediated mechanism, activated EGF receptor and contributed to H4 cell proliferation in a paracrine manner.

**Conclusions:**

Our results show that Rab27b mediates the radioresistance of highly malignant GBM cells. Rab27b promotes the proliferation of adjacent cells through EREG-mediated paracrine signaling after irradiation. Thus, the Rab27b-EREG pathway is a novel potential target to improve the efficacy of radiotherapy in GBM.

**Key Points**

- The Rab27b pathway can be targeted to improve the efficacy of radiotherapy in GBM.
- Co-upregulation of Rab27b and EREG after ionizing radiation contributes to radioresistance in GBM.
- Rab27b mediates EREG secretion, which leads to paracrine signaling after ionizing radiation.

**Importance of the Study**

Glioblastoma (GBM) is the most aggressive brain tumor, and patients with GBM have an extremely poor prognosis. Although radiotherapy combined with chemotherapy after surgery prolongs the overall survival of patients with GBM, tumor resistance to treatment leads to recurrence. In many cases, cancer cells release secretory factors through vesicle trafficking that leads to resistance to treatment. However, the roles and the molecular signaling pathways by which these secretory factors mediate radioresistance remain unknown. Here, we show that Rab27b is upregulated in GBM cells after ionizing radiation treatment. Rab27b knockdown enhances the cytotoxicity of radiotherapy in GBM in vitro and in an orthotopic tumor model in vivo. In addition, Rab27b regulates the expression of EREG in U87MG GBM cells, which further promotes the proliferation of adjacent cells through paracrine effects. Taken together, Rab27b signaling can be targeted to improve the efficacy of radiotherapy in GBM.

Glioblastoma (GBM) is a highly aggressive brain tumor with a very poor prognosis. Adjuvant radiotherapy has been shown to improve disease outcomes, but the recurrence rate remains high, resulting in a poor prognosis with a 5-year survival rate less than 7%.^1^

Intercellular communication mediated by radiation-induced secreted proteins in the tumor microenvironment has been implicated in the radioresistance of cancer cells and the promotion of tumor progression.^2–4^ However, the regulation of secreted factors after radiation treatment in GBM and its relation to radioresistance have not been elucidated. Thus, the identification of the mechanisms that regulate protein secretion through vesicle transport after ionizing radiation (IR) treatment is important for improving GBM treatment.

Rab27b, a member of the Rab family, has been reported to promote tumor progression and metastasis.^5–7^ Rab27b regulates the extracellular trafficking of vesicles and the release of secreted factors.^8,9^ The secreted factors that are released by cancer cells have important roles in promoting cancer cell proliferation, survival, invasion, and remodeling of the tumor microenvironment.^10,11^ Epiregulin (EREG), a member of the epidermal growth factor (EGF) family, is a secreted factor that is released to the extracellular region and induces changes to nearby cells, that is, exerts paracrine effects.^12^

According to a previous study, the expression of the *RAB27B* mRNA is increased in MCF-7 breast cancer cells after IR exposure.^13^ However, the role of Rab27b in the radioresistance of GBM cells has not been elucidated. In the present study, we show that Rab27b expression is significantly upregulated after IR treatment and plays a crucial role in radioresistance in GBM both in vitro and in vivo. Furthermore, Rab27b regulates the expression of EREG and further participates in paracrine signaling by activating EGF receptor (EGFR) in different types of glioma cells after IR treatment. Our study provides a potential strategy to improve the efficacy of radiotherapy in GBM by inhibiting the Rab27b–EREG pathway.

## Materials and Methods

### Cell Culture

The human brain cell lines H4, SW1088, A172, U118MG, and U87MG were purchased from the American Type Culture Collection. The detail is provided in Supplementary Data.

### Irradiation

For the in vitro study, cells were irradiated with 130 kV of X-rays using a CellRad X-ray generator (Precision). For the in vivo study, whole brains of tumor-bearing mice were irradiated with 150 kV of X-rays (HITACHI). Irradiation was performed at doses of 2, 4, 6, or 8 Gy, according to the experiments.

### Microarray Analysis

U87MG cells in three-dimensional laminin-rich extracellular matrix (3D lrECM) were irradiated with a daily fraction of 4 Gy for 4 days. Total RNA was extracted from U87MG cells using a NucleoSpin RNA kit (Macherey-Nagel). A High-Sensitivity 3D-Gene Human Oligo chip 25k version 2.10 (Toray Industries) was used for the microarray analysis. The data were normalized to the corresponding data from the untreated group by Toray Industries.

### RNA Isolation and Real-Time PCR

Total RNA was extracted with TRI reagent (Thermo Fisher Scientific) and cDNAs were synthesized using the SuperScript IV First-Strand Synthesis System (Thermo Fisher Scientific). Real-time PCR was performed with Light Cycler 96 (Hoffman-La Roche Ltd) using FastStart Essential DNA Green Master kit (Hoffman-La Roche Ltd). The sequences of primers are described in Supplementary Table 1.

### Cell Lysis and Western Blotting

Briefly, cells were lysed with radioimmunoprecipitation assay buffer. Proteins were separated by gel electrophoresis and then transferred to an Immobilon-FL polyvinylidene fluoride membrane (Merck Millipore). After blocking with Odyssey buffer (LI-COR Biosciences), the membrane was incubated with a primary antibody. The membrane was washed and incubated with a secondary antibody and then the fluorescence of the secondary antibody was detected. A detailed description of the procedure, reagents, and antibodies is provided in Supplementary Data.

### Immunofluorescence Staining

Briefly, cells were fixed with 4% paraformaldehyde and permeabilized with 0.2% Triton X-100 in phosphate-buffered saline (PBS). After blocking with 5% bovine serum albumin (BSA) in PBS, cells were incubated with a primary antibody. Then, cells were incubated with a secondary antibody. Filamentous actin was stained with phalloidin (Thermo Fisher Scientific) and the cells were incubated with 4′,6′-diamidino-2-phenylindole. A detailed description of the procedure, reagents, and antibodies is provided in Supplementary Data.

### Apoptosis Assay

An Annexin V-FITC Apoptosis Detection Kit (Abcam) was used to analyze apoptosis. Fluorescence was measured using a FACSAria III flow cytometer (BD Biosciences). The detailed procedure is provided in Supplementary Data.

### Small Hairpin RNA and Transfection

Small hairpin RNAs (shRNAs) flanked by 5′ and 3′ arms of the miR-30 precursor were subcloned into a piggyBac transposon-based vector pPB CEH MCS IP, together with the 5′ flanking mTagBFP2 cDNA sequence.^14^ Resulting vectors were stably integrated into the genome of U87MG cells by co-transfection with a piggyBac transposase expression vector. The target sequences are described in Supplementary Table 1. Oligo DNAs for the target sequences were purchased from Thermo Fisher Scientific. Transfection was performed using ViaFect Transfection Reagent (Promega) according to the manufacturer’s instructions.

### Small Interfering RNAs and Transfection

The small interfering RNA (siRNA) sequences are described in Supplementary Table 1. Transfection was performed using Lipofectamine RNAiMAX Transfection Reagent (Thermo Fisher Scientific). A detailed description of the procedure is provided in Supplementary Data.

### Cell Viability Assay

Cells were treated with 8 Gy × 2 times irradiation. After 72 h, cells were harvested with trypsin-EDTA (Nacalai Tesque) and counted. Cell numbers were normalized to the untreated group to calculate the relative cell viability.

### Colony Formation Assay

Cells were seeded in 6-well plates and irradiated 24 h later. Fourteen days after IR exposure, cells were fixed with 4% paraformaldehyde for 10 min at room temperature. Colonies were then stained with 1% crystal violet (Sigma-Aldrich). The surviving fraction was calculated by normalizing the data to the plating efficiency.

### In Vivo Experiment

For the orthotopic mouse model, U87MG-Luc cells transfected with shControl (shCTL) or shRab27b were injected into the brains of female BALB/c-nu/nu nude mice. Tumor-bearing mice were divided into 2 groups: a sham-irradiated control group and a group treated with 4 Gy × 4 times whole-brain fractionated irradiation. The bioluminescence of the mice was measured using an in vivo imaging system (IVIS Spectrum CT; PerkinElmer). Mice were sacrificed and the brains were obtained when the mice prostrated. Rab27b and EREG expressions in brain tumors were analyzed by immunohistochemistry (IHC). All animal studies were approved by the Institutional Animal Care and Use Committee of Hokkaido University (#16-0137). The detailed procedure is provided in Supplementary Data.

### The Cancer Genome Atlas Analysis

RNA-seq (*n* = 160, RSEM) or microarray (*n* = 206, RSEM, *Z*-score) data, together with corresponding clinical data from patients with GBM, were obtained from The Cancer Genome Atlas (TCGA). Patients were stratified by *RAB27A* or *RAB27B* expression; the top 25% and the remaining patients were categorized into “*RAB27A/RAB27B*-high” and “*RAB27A/RAB27B*-low” groups, respectively. Patients characterized as both *RAB27A*-high and *RAB27B*-high (*RAB27A/RAB27B*-high/high) were also compared to the other patient groups. Survival curves were estimated using the Kaplan–Meier method and compared by log-rank tests. The same classifications were also used to determine correlations with the average expression level of *EREG*. Statistical significance was assessed using the Brunner–Munzel test, which is independent of the distributions and variances.

### Paracrine Assay

The medium of H4 cells was replaced with FBS-free medium. After incubation for 16 h, conditioned medium from U87MG cells cultured for 24 h was added to the H4 cells. After 10 min, H4 cells were lysed and analyzed using western blotting to measure the levels of phosphorylated EGFR.

### Coculture

Cell culture inserts with a pore size of 0.4 µm (Millicell) were used for 2D coculture. For 3D coculture, H4 or U87MG cells layer were sandwiched with lrECM layers, on which H4-Luc cells were seeded. The detail is provided in Supplementary Data.

### Statistical Analysis

The in vitro experiments were repeated at least 3 times. Unpaired 2-tailed *t*-tests were used to calculate *P* values. *P* value less than .05 was considered statistically significant.

## Results

### Rab27b Expression Is Upregulated by IR in U87MG GBM Cells

**Figure 1.**
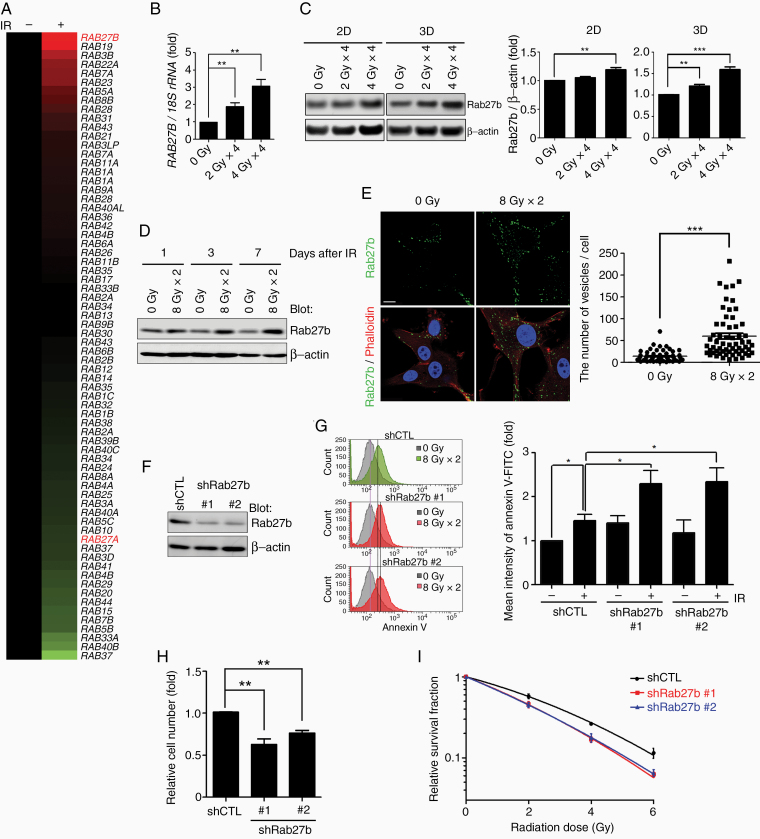
Rab27b expression is increased by IR treatment and is involved in the radioresistance of GBM U87MG cells. (A) The expression of Rab family genes in U87MG cells cultured in 3D lrECM was analyzed using a cDNA microarray. The heatmap shows increased (red) or decreased (green) RNA levels in 4 Gy × 4 IR-treated U87MG cells compared to untreated control cells. (B) The relative levels of the *RAB27B* mRNA were measured in 3D lrECM-cultured U87MG cells after fractionated irradiation. Real-time PCR was conducted to determine the mRNA level of *RAB27B*. *RAB27B* levels were normalized to *18S rRNA* levels. Columns, mean (*n* = 4). (C) The relative protein levels of Rab27b were measured after fractionated irradiation in 2D or 3D lrECM-cultured U87MG cells by western blotting. The band intensities of Rab27b were normalized to β-actin. Columns, mean (*n* = 3). (D) The relative protein levels of Rab27b were measured after 8 Gy × 2 irradiation in 2D-cultured U87MG cells. Lysates were collected 1, 3, and 7 days after IR treatments. (E) Images of Rab27b immunofluorescence in U87MG cells were acquired using confocal microscopy after IR treatment. Cells were fixed and stained with an anti-Rab27b antibody and phalloidin 24 h after IR treatment. Green: Rab27b, red: filamentous-actin (phalloidin), blue: nuclei (4′,6′-diamidino-2-phenylindole [DAPI]). Scale bar, 10 µm. The number of Rab27b-positive vesicles per cell was counted. Columns, mean (*n* > 60 cells). (F) Rab27b knockdown was induced by shRNAs (shRab27b #1 and #2) in U87MG cells. Rab27b protein levels were confirmed using western blotting. (G) Apoptotic cells were identified using Annexin V-FITC staining followed by flow cytometry. The mean fluorescence intensity of FITC was calculated and normalized to the untreated control group. Columns, mean (*n* = 4). (H) Rab27b knockdown (shRab27b #1 or #2) **Figure 1. Continued** or control (shCTL) cells were exposed to 8 Gy × 2 IR. Relative cell numbers were determined by cell counting and normalized to the control group. Columns, mean (*n* = 3). (I) The relative survival of shCTL or shRab27b U87MG cells after IR treatment was analyzed using a clonogenic survival assay and normalized to the nonirradiated group. Cells were fixed 15 days after seeding and stained with crystal violet. Points, mean (*n* = 3). All graphs indicate the means ± SE; *P < .05, **P < .01, ***P < .001.

Rab GTPases regulate elements of vesicular trafficking, including exocytosis and protein release, which leads to therapeutic resistance through the remodeling of the tumor microenvironment and its effect on adjacent cells.^8,15–17^ We hypothesized that specific Rab proteins play important roles in radioresistance in high-grade glioma. We first performed a microarray analysis to screen the mRNA expression levels of Rab family members in U87MG GBM cells after irradiation. According to the comprehensive analysis, the level of the *RAB27B* mRNA was increased to the greatest extent among Rab family members after 4 Gy × 4 irradiation in 3D lrECM-cultured U87MG cells compared to nonirradiated cells, as confirmed by real-time PCR (Figure 1A and B). The level of the Rab27b protein was increased by 4 Gy × 4 irradiation in both 2D and 3D lrECM-cultured U87MG cells (Figure 1C). We further confirmed that Rab27b expression was increased by fractionated irradiation at a different dose (8 Gy × 2) in 2D-cultured U87MG cells. Increased expression of Rab27b was sustained after 1 week of irradiation (Figure 1D). Consistent with the western blot results, immunofluorescence images showed an increased number of Rab27b-positive vesicles in U87MG cells after IR treatment (Figure 1E). Notably, the expression of Rab27a, another member of the Rab27 subfamily, was not increased at the protein level in U87MG cells after IR treatment, as confirmed by western blot analysis after validation of the Rab27a-specific antibody (Supplementary Figure S1A and B). We also verified the levels of the Rab27b protein after IR treatment in the U118MG cells in addition to U87MG cells (Supplementary Figure S2A). Thus, among the Rab family proteins, Rab27b is specifically upregulated in GBM cells in response to IR treatment.

### Rab27b Knockdown Enhances the Response of U87MG Cells to Radiation

We previously showed that proteins upregulated after IR can contribute to the survival of cancer cells.^18,19^ We performed shRNA-mediated knockdown of Rab27b in U87MG cells to determine the role of Rab27b in radioresistance (Figure 1F). We stained the cells with Annexin V to analyze apoptosis in Rab27b knockdown cells after IR. Compared to the control group of U87MG cells, the Rab27b knockdown group exhibited increased apoptosis after IR treatment (Figure 1G). Furthermore, Rab27b knockdown effectively reduced the cell number and suppressed clonogenic cell survival (Figure 1H and I), indicating a decreased ability to form a large colony after IR treatment. The same effect was confirmed in U118MG cells (Supplementary Figure S2B). These results suggest that Rab27b suppression might increase the effectiveness of IR by reducing the viability of U87MG cells.

### Rab27b Knockdown Increases the Effect of IR In Vivo

**Figure 2.**
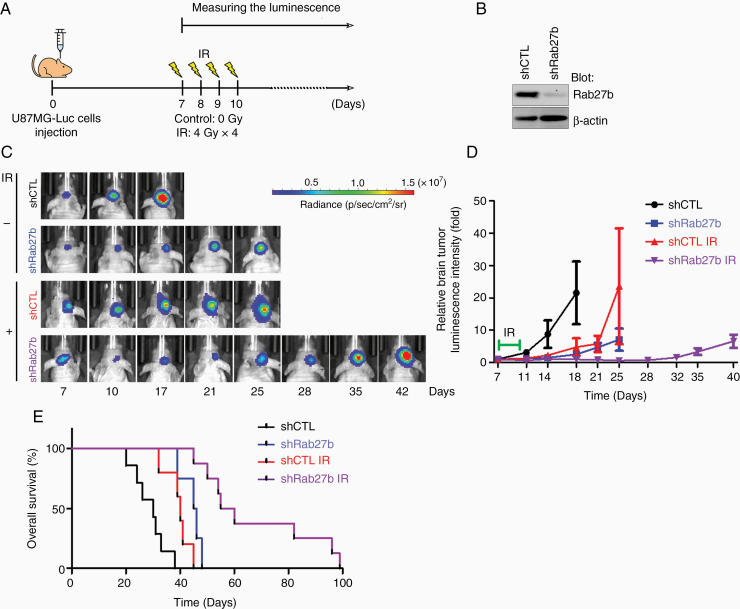
Rab27b knockdown improves the effect of radiation on GBM tumor growth and prolongs the overall survival of mice. (A) Experimental scheme. U87MG-Luc shCTL or shRab27b tumor-bearing mice (n = 6-9) received whole-brain irradiation from days 7 to 10 after cells were injected. (B) Knockdown of Rab27b by shRNA in U87MG-Luc cells was confirmed by western blotting. (C) Tumor growth was monitored by measuring the luminescence using in vivo imaging system until mouse prostration. (D) The luminescence intensity in tumors was analyzed and normalized to the intensity measured on day 7. (E) Kaplan–Meier survival curves.

We next validated the in vivo effect of Rab27b knockdown on radioresistance in an orthotopic xenograft mouse model using the experimental scheme shown in Figure 2A. U87MG-Luc cells, which stably expressed redshifted *Luciola italica* luciferase, were generated to analyze tumor growth by measuring the luminescence intensity. U87MG-Luc cells were further transfected with plasmids carrying a control shRNA sequence (shCTL) or the Rab27b #1 shRNA sequence (shRab27b; Figure 2B). Rab27b knockdown alone was effective at delaying tumor growth and prolonging the survival of mice. Importantly, IR treatment in combination with Rab27b knockdown exerted synergistic effects that were greater than the addition of the effect of each treatment on each group (Figure 2C–E). Taken together, these in vivo results strongly suggest that Rab27b contributes to radioresistance in GBM.

### Feedback Upregulation of Rab27b and EREG in GBM Cells

**Figure 3.**
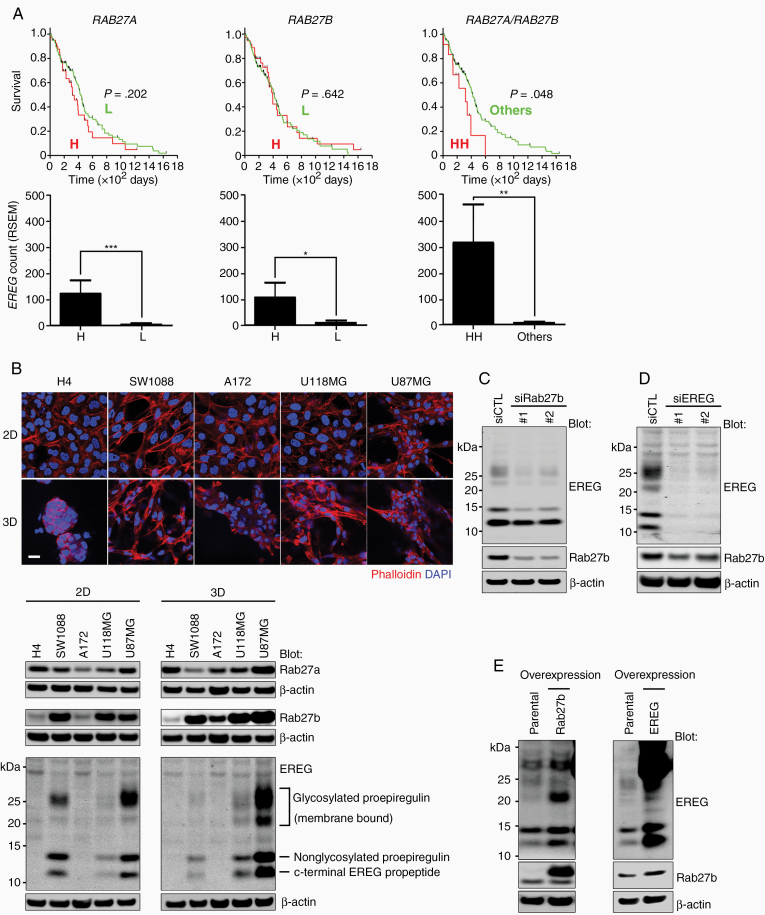
The expression of both Rab27b and EREG is co-upregulated in highly malignant glioma cell lines. (A) Kaplan–Meier survival curves (upper panels) were generated for patients stratified by *RAB27A* or *RAB27B* expression either alone or in combination using TCGA RNA-seq data from 160 human patients with GBM. The average values of EREG counts in each group were also calculated (lower panels). H, high; L, low; HH, *RAB27A/RAB27B*-high/high Columns, mean; bars, SE; *P < .05, **P < .01, ***P < .001, Brunner–Munzel test. (B) Images of immunofluorescence (upper panels) showing the morphology of 5 glioma cell lines—H4, SW1088, A172, U118MG, and U87MG—in 2D or 3D lrECM culture. Red: filamentous-actin (phalloidin), blue: nuclei (DAPI). Scale bar, 25 µm. The protein levels of Rab27b and EREG in different glioma cell lines were analyzed by western blotting (lower panels). (C) EREG expression was analyzed in lysates of U87MG cells transfected with siRab27b or siCTL. (D) Rab27b expression was measured in U87MG cells transfected with siEREG or siCTL. (E) Rab27b and EREG levels were measured in lysates from U87MG cells stably overexpressing Xpress-Rab27b or EREG-Venus using western blotting.

Rab27b has been reported to regulate vesicle trafficking for the exocytosis of growth factors to activate their receptors.^20^ Although these effects might largely be mediated by the regulation of trafficking processes, we sought to identify secretory proteins whose transcription correlated with Rab27b. We explored the mRNA levels of secreted proteins based on microarray data and TCGA analyses of GBM. Among these proteins, we focused on EREG because its expression was increased after IR treatment and correlated with Rab27b expression, as confirmed by the microarray and TCGA data, respectively. EREG has been implicated in glioma progression and tumor growth.^21,22^ The TCGA analysis suggested a correlation between the individual expression level of *RAB27A* or *RAB27B* with *EREG* expression. Notably, increased expression of both *RAB27A* and *RAB27B* correlated with a poor prognosis. In tumors with high expression of both *RAB27A* and *RAB27B*, the average *EREG* expression level was significantly higher than in the group with low *RAB27A* and *RAB27B* expression (Figure 3A). Similar results were obtained in another dataset (Supplementary Figure S3). Thus, increased expression of EREG is mediated by both Rab27a and Rab27b, although only the latter is upregulated in response to IR.

To further evaluate the expression of EREG and Rab27b in glioma cell lines, we analyzed the protein levels in the H4 (neuroglioma), SW1088 (astrocytoma), A172 (GBM), U118MG (GBM), and U87MG (GBM) cells. H4 cells, low-grade glioma cells, showed an epithelial-like phenotype in 2D culture, while the other cell lines exhibited fibroblast-like phenotypes in both 2D and 3D cultures. The EREG protein was expressed at higher levels in the U87MG GBM cell line, which expressed both Rab27a and Rab27b at high levels, than in H4 neuroglioma cells in both 2D and 3D cultures (Figure 3B). EREG is initially synthesized as a transmembrane proepiregulin protein that is then processed to produce a C-terminal propeptide and a mature form of EREG. Mature EREG is released to the extracellular region and binds to EGFRs, leading to their activation.^21,23^ The expression pattern of EREG was closely correlated with Rab27b, but not Rab27a, in the 5 tested glioma cell lines, and EREG was substantially upregulated in U87MG cells. We then analyzed the regulation of EREG and Rab27b in U87MG cells. Rab27b knockdown decreased the protein level of EREG in U87MG cells (Figure 3C). Conversely, EREG knockdown reduced the protein level of Rab27b (Figure 3D). Exogenous overexpression of Rab27b increased EREG expression, and exogenous overexpression of EREG increased Rab27b expression in U87MG (Figure 3E) and U118MG cells (Supplementary Figure S2C). These results suggest a positive feedback loop that regulates Rab27b and EREG expression.

### EREG Is Involved in Radioresistance

**Figure 4.**
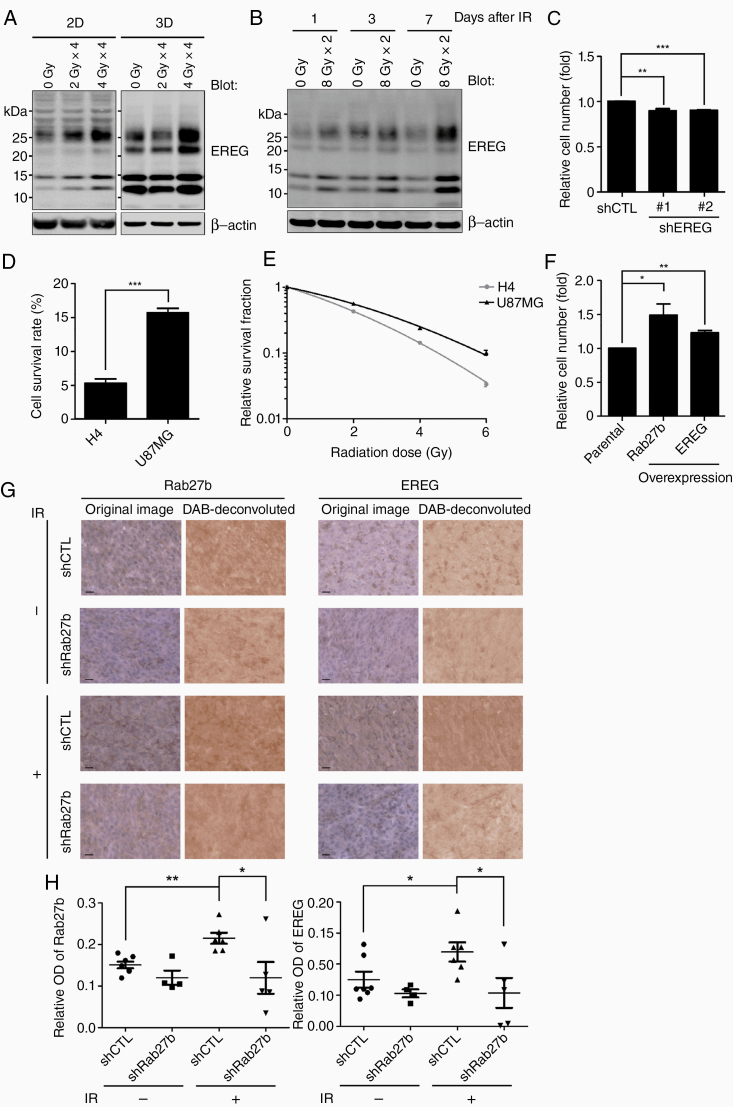
Upregulation of EREG after IR is involved in the radioresistance of GBM cells. (A) The protein levels of EREG were analyzed in U87MG cells after IR treatment using western blotting. (B) The relative protein levels of EREG were measured after fractionated irradiation of 2D-cultured U87MG cells. Lysates were collected 1, 3, and 7 days after IR treatments. (C) The relative cell numbers were determined by counting the shEREG or shCTL cells after 8 Gy × 2 IR. The relative cell numbers were normalized to the nonirradiated groups. (D) The viability of H4 and U87MG cells was measured by counting the cells after 8 Gy × 2 IR. The relative cell numbers were normalized to the nonirradiated groups. (E) Clonogenic survival of H4 and U87MG cells after IR treatment, as normalized to the nonirradiated groups. Cells were fixed 15 days after seeding and stained with crystal violet. (F) Xpress-Rab27b or EREG-Venus was stably overexpressed in H4 cells. Cell viability after 8 Gy × 2 IR was analyzed by cell counting. (C–F) Columns or points, mean (*n* = 3). All graphs indicate the means ± SE; *P < .05, **P < .01, ***P < .001. (G) Representative images of IHC staining with Rab27b or EREG. The images were color deconvoluted to 3,3′-Diaminobenzidine (DAB) images using Fiji. The bars indicate 20 µm. (H) The optical density (OD) was quantified using Fiji. The relative OD was calculated as follows: (the OD of a DAB image with a primary antibody staining) – (the OD of a DAB image with negative staining). Columns, mean with SE; shCTL (*n* = 7), shRab27b (*n* = 4), shCTL IR (*n* = 6), shRab27b IR (*n* = 5); *P < .05, **P < .01.

Consistent with the IR-induced upregulation of Rab27b shown in Figure 1, the protein level of EREG was increased after IR treatment in U87MG (Figure 4A) and U118MG cells (Supplementary Figure S2A). The increase in EREG expression persisted for at least 1 week after irradiation (Figure 4B). EREG knockdown led to a modest decrease in the cell number of U87MG cells exposed to IR (Figure 4C). The relative cell viability rate assessed by counting cells and the clonogenic survival fraction were higher in U87MG cells, which exhibited co-upregulation of Rab27b and EREG, than in H4 cells (Figure 4D and E). Exogenous overexpression of either Rab27b or EREG contributed to radioresistance in relatively radiation-sensitive H4 cells (Figure 4F). In contrast, overexpression of these proteins in U87MG cells did not lead to a further increase in radioresistance (Supplementary Figure S4). We then confirmed the increased protein levels of Rab27b and EREG in mouse brain tumors after IR treatment by IHC staining. Importantly, Rab27b knockdown suppressed not only Rab27b but also the IR-induced EREG, indicating the co-regulation of both proteins after IR (Figure 4G and H).

These data suggest that IR induces the co-upregulation of Rab27b and EREG, which contribute to radioresistance in glioma cells.

### Secretion of EREG via Rab27b in U87MG Cells Promotes the Proliferation of Recipient H4 Cells in a Paracrine Manner

**Figure 5.**
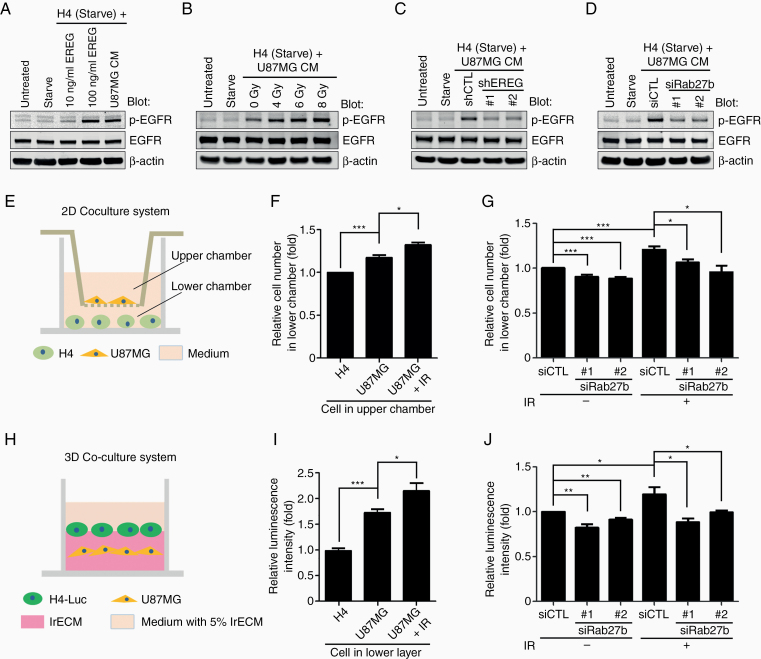
EREG secreted through the Rab27b pathway in U87MG cells stimulates EGFR activation and the proliferation of recipient H4 cells in a paracrine manner. (A–D) The phosphorylation of EGFR in H4 cells was assessed to analyze the paracrine effect. H4 cells were preincubated with serum-free medium for 16 h and then stimulated with recombinant EREG or conditioned medium from U87MG cells for 10 min, as indicated. EGFR phosphorylation was analyzed by western blotting using an antibody against p-EGFR (Tyr1068). (A) H4 cells were stimulated with 10 or 100 ng/mL EREG or with conditioned medium from U87MG cells. (B) H4 cells were stimulated with conditioned medium from U87MG cells collected 24h after IR treatments at the indicated doses. (C) EREG knockdown was mediated by shRNAs (shEREG #1 or #2) in U87MG cells. H4 cells were stimulated with conditioned medium from EREG knockdown cells or control (shCTL) U87MG cells. (D) H4 cells were stimulated with conditioned medium from U87MG cells transfected with siRab27b or siCTL, as indicated. (E) An experimental model of the 2D coculture system. H4 or pretreated U87MG cells were placed in the Transwell insert (upper chamber). U87 cells were pretreated with siRNA for 24 h and/or IR. The upper chambers were placed in the wells of 6-well plates seeded with H4 cells (lower chambers). After 3 days of coculture, the number of H4 cells in the lower chambers was counted. (F) The numbers of H4 cells cocultured with H4, U87MG, or 8 Gy IR-treated U87MG cells were counted and normalized to H4 cells cocultured with H4 cells. Columns, mean (*n* = 4). (G) The number of H4 cells cocultured with U87MG cells transfected with siRNA followed by IR was counted and normalized to H4 cells cocultured with control cells. Columns, mean (*n* ≧ 5). (H) An experimental model of the 3D coculture system. H4 or U87MG cells treated with siRNA were seeded. After incubation for 24 h, these cells were covered with an additional lrECM layer on which H4-Luc cells were seeded. (I) The luminescence intensity of luciferase in H4-Luc cells cocultured with H4, U87MG, or 8 Gy IR-treated U87MG cells was measured and normalized to H4-Luc cells cocultured with H4 cells. Columns, mean (*n* = 5). (J) The luminescence intensity of luciferase in H4-Luc cells cocultured with U87MG cells transfected with siRNA followed by IR was measured and normalized to H4-Luc cells cocultured with control cells. Columns, mean (*n* = 4). All graphs indicate the means ± SE; *P < .05, **P < .01, ***P < .001.

Gliomas exhibit genetic heterogeneity at the intratumoral level, and proteins secreted from cancer cells potentially affect adjacent cells.^24,25^ EREG, a secreted protein, has been reported to be involved in the paracrine regulation of tumor progression.^26,27^ Accordingly, we assessed whether EREG secretion by U87MG cells through a mechanism mediated by the Rab27b pathway after IR treatment exerts paracrine effects on different types of glioma cells, particularly the low-grade, radiation-sensitive H4 cell line. Proepiregulin is processed to yield a membrane-bound C-terminal peptide and a mature form that is released into the extracellular space.^23^ Mature EREG binds to EGFR and activates EGFR signaling.^21^ We hence examined the paracrine effect by assessing the level of phosphorylated EGFR (p-EGFR) in H4 cells treated with recombinant EREG or conditioned medium from U87MG cells. Similar to the effect of recombinant EREG, the p-EGFR level increased in H4 cells treated with conditioned medium from U87MG cells (Figure 5A). Moreover, EGFR exhibited a dose-dependent increase in phosphorylation in H4 cells cultured with medium from IR-treated U87MG cells compared to medium from nonirradiated cells (Figure 5B). A lower p-EGFR level was observed in cells treated with conditioned medium from EREG knockdown U87MG cells than in cells incubated with conditioned medium from control cells (Figure 5C). Similar results were obtained in cells treated with conditioned medium from Rab27b knockdown U87MG cells (Figure 5D). Thus, secreted EREG, which is regulated by Rab27b in response to IR treatment of U87 MG cells, activates EGFR in H4 cells.

We further confirmed the paracrine effects of IR-treated U87MG cells on the proliferation of H4 cells using both 2D (Figure 5E) and 3D coculture systems (Figure 5H). H4 cells cocultured with U87MG cells exhibited increased proliferation compared to those cocultured with H4 cells. Furthermore, H4 cells cocultured with IR-treated U87MG cells exhibited increased proliferation compared to nonirradiated cells (Figure 5F). In contrast, the increase in H4 cell proliferation was suppressed by coculture with Rab27b knockdown cells compared to coculture with control U87MG cells after IR treatment (Figure 5G). Similar results were obtained in the 3D coculture system, as evaluated by measuring the luminescence intensity of H4-Luc cells (Figure 5I and J). Collectively, our data suggest that after IR treatment, the Rab27b–EREG pathway increases proliferation by exerting a paracrine effect.

## Discussion

Radiotherapy in combination with surgery and chemotherapy is the most commonly prescribed treatment for GBM, but the prognosis of patients with GBM remains poor due to therapeutic resistance and tumor recurrence.^1,28^ Here, we show that the radiation-induced increase in Rab27b-EREG signaling contributes to radioresistance in GBM cells and further exerts pro-proliferative paracrine effects on surrounding cells.

Rab27b promotes malignant cell phenotypes and is associated with poor patient prognosis in several cancers.^5–7,29,30^ Our study shows that Rab27b expression was increased after irradiation and was sustained for 7 days, supporting the findings that Rab27b expression is increased at several endpoints of assays and experiments, such as the cell counting assay, colony formation assay, paracrine assay, and in vivo experiments. Silencing of Rab27b decreased GBM cell survival after IR treatment in vitro and further delayed tumor growth and increased overall survival after radiation treatment in an in vivo model. In contrast to our results, Huang et al.^31^ reported that Rab27b suppression increased nasopharyngeal cancer cell survival after IR treatment. The difference between the results of the previous study and our results suggests that the role of Rab27b in the radiation response may differ, depending on the type of cancer.

The interaction and invasion of the tumor cells to surrounding tissues are crucial for tumor progression.^32^ Rab27b has been reported to mediate intracellular communication via secreted factors that promote cell proliferation, invasion, and tumor growth.^8,9,20,22,26^ In our in vivo experiment, Rab27b knockdown delayed tumor growth in mice after IR treatment. These results suggest that Rab27b might also play important roles in invasion, the interaction with surrounding tissues as well as cell proliferation after irradiation. The roles and molecular mechanisms of Rab27b in mediating radioresistance and tumor growth after irradiation should be investigated in more detail in future studies.

Rab27a and Rab27b share 71% amino acid homology.^33^ Both Rab27a and Rab27b mediate the release of RANKL, the receptor activator of NF-kB ligand, in osteoblastic cells^20^; in addition, both are associated with melanosomes in melanocytes, suggesting that these 2 proteins are functionally homologous.^33^ On the other hand, Rab27b expression is thought to show a more restricted pattern than Rab27a.^34^ Rab27a and Rab27b are differentially expressed and affect the secretion of different peptide hormones in pituitary cells,^9^ suggesting that they have different functions. In HeLa cells, Rab27a and Rab27b control different steps in the process of exosome secretion.^8^ In our study, the expression patterns of Rab27a and Rab27b differed among glioma cell lines. In addition, Rab27b was specifically increased in IR-exposed GBM cells, but Rab27a expression was not increased. Thus, the mechanisms regulating Rab27a and Rab27b might differ in response to IR treatment in GBM cells.

Cancer cells communicate with each other and their microenvironment by releasing growth factors, cytokines, proteases, and exosomes to promote their survival and progression.^35,36^ To our knowledge, this study is the first to indicate that EREG expression is upregulated by IR treatment and is involved in radioresistance in cancer. Our results show that the decrease in radioresistance caused by Rab27b knockdown was higher than that caused by EREG knockdown. As a possible reason, Rab27b regulates the secretion of not only EREG but also other factors. EREG partially contributes to radioresistance and other factors also may be upregulated after IR and mediate radioresistance via enhanced Rab27b.

EREG is synthesized as a glycosylated proepiregulin protein that is processed to produce a C-terminal propeptide and a mature form.^23^ Interestingly, Figures 3B and 4A show different patterns of protein bands pattern in 2D and 3D cultures. In particular, the levels of the 2 protein bands at approximately 12 kDa and 22 kDa were higher in 3D-cultured cells than in 2D-cultured cells. The 12 kDa band is the C-terminal EREG propeptide. The processing of proepiregulin is mediated by a disintegrin and metalloprotease (ADAM) 17.^37^ Thus, the activity of ADAM17 may be different in cells cultured under 2D and 3D conditions, resulting in a difference in the level of the C-terminal EREG propeptide. The 22 kDa band corresponds to glycosylated proepiregulin. Protein glycosylation is sensitive to the environment of cells.^38^ Because the cell environment is substantially different between 2D plastic and 3D lrECM, EREG glycosylation may be affected by the cell culture conditions.

In the present study, we found that Rab27b regulated EREG expression and secretion after irradiation and that the secreted EREG protein also affects Rab27b upregulation. Although we show that the expression of Rab27b and EREG was reciprocally regulated through a feedback loop, the underlying mechanisms are not fully understood. Several cytokines, such as IL-6 and IL-17, are reported to increase EREG expression, which activates intracellular signaling cascades, including the NF-κB, STAT3, and MAPK cascades.^39,40^ Moreover, in addition to EREG, other soluble proteins may be involved in the EREG–Rab27b feedback loop after the IR stimulation of GBM. Further studies are needed to understand the mechanisms by which Rab27b and EREG are upregulated by IR treatment and further interact with each other.

GBM tumors are composed of a heterogeneous combination of cells with distinct phenotypes and proliferative potential, which contributes to the therapeutic resistance of GBM.^25,41^ Our data suggest that Rab27b may contribute to GBM growth by promoting the proliferation of other subpopulations of intratumoral cells through EREG secretion after IR treatment. Coculture with highly malignant U87MG cells increased the proliferation of low-malignant H4 cells. This paracrine effect was enhanced by the IR treatment of U87MG cells. Thus, the secretion of soluble factors through the Rab27b pathway after radiation potentially alters cancer cell subtypes and/or induces transdifferentiation in the GBM tumor microenvironment.

Our data suggest that Rab27b-silencing radiosensitized GBM cells. Two main steps are necessary for the translation of agents targeting Rab27b into the clinic. First, an inhibitor that blocks the interaction of Rab27b with its effector must be designed. An inhibitor of Rab27a, a homolog of Rab27b, has already been developed and is commercially available. The Rab27a inhibitor Nexinhib20 inhibits the binding of Rab27a to synaptotagmin-like protein 1, a Rab27a effector.^42^ Inhibitors for Rab27b may be developed using a similar strategy as Rab27a inhibitors. Second, a delivery system that overcomes blood–brain barrier to transport the Rab27b inhibitor into the tumor region is also needed. Recently, several strategies have been studied to deliver agents through the blood–brain barrier, such as nanoparticles and exosomes.^43^ The development of these research areas would overcome the challenge of delivering therapeutic agents into the brain. Multifaceted research is needed for translation to clinical treatment.

In conclusion, we found that Rab27b and EREG are co-upregulated in highly aggressive brain cancer cells, and their expression is further increased after IR treatment, leading to radioresistance in GBM. In addition, Rab27b regulates EREG in response to the IR treatment, and EREG interacts with adjacent cells to promote tumor progression. Based on these data, the Rab27b–EREG pathway might be targeted to improve the efficacy of radiotherapy in GBM.

## Supplementary Material

vdaa091_suppl_Supplementary_Material

## Notes

**Authorship Statement.** Conception and design: S.N., P.-H.W., Y.O., and J.-M.N. Data acquisition, analysis, and interpretation: S.N., P.-H.W., Y.O., and J.-M.N. Development of the methodology and material support: S.N., P.-H.W., T.Y., A.J.G., Q.-T.L., H.S., S.S., Y.O., and J.-M.N. Writing of the manuscript: S.N., P.-H.W., H.A., Q.-T.L., Y.O., and J.-M.N. Supervision: Y.O. and J.-M.N.
