# Supplementary material for: Rab27b contributes to radioresistance and exerts a paracrine effect via epiregulin in glioblastoma
Source: Neurooncol Adv. 2020 Aug 8;2(1):vdaa091. doi: 10.1093/noajnl/vdaa091 (PMC7770522; doi:10.1093/noajnl/vdaa091)
Supplement: vdaa091_suppl_Supplementary_Material [file vdaa091_suppl_supplementary_material.docx]

**Supplementary Data**

**Supplementary Materials and Methods**

**Cell Culture**

The human brain cell lines H4, SW1088, A172, U118MG and U87MG were purchased from the American Type Culture Collection. H4, A172 and U118MG cells were cultured in Dulbecco’s Modified Eagle’s Medium (Sigma-Aldrich) containing 10% fetal bovine serum (FBS; GE Healthcare Life Sciences) at 37°C with 5% CO_2_. SW1088 cells were cultured in L-15 Medium (Thermo Fisher Scientific) containing 10% FBS at 37°C with atmospheric air. U87MG cells were cultured in Minimum Essential Medium Eagle (Sigma-Aldrich) containing 10% FBS at 37°C with 5% CO_2_. For the *in vivo* imaging study, U87MG-Luc cells stably expressing redshifted *Luciola italica* luciferase (Red-FLuc) were produced. U87MG cells were infected with the Red-FLuc-expressing lentivirus and selected with 10 µg/ml blasticidin S (Thermo Fisher Scientific).

**Three-Dimensional LrECM Culture**

For three-dimensional laminin-rich extracellular matrix (3D lrECM) cultures, cells were seeded on top of growth factor-reduced Basement Membrane Extract (Corning) as described previously.^1^

**Cell Lysis and Western Blotting**

Cells were lysed with radioimmunoprecipitation assay (RIPA) buffer (1% NP-40, 150 mM NaCl, 50 mM Tris-HCl [pH 7.4], 5 mM ethylenediaminetetraacetic acid [EDTA], 1% sodium deoxycholate, 0.1% sodium dodecyl sulfate [SDS] 1 mM Na_3_VO_4_, 1 mM NaF and a protease inhibitor cocktail [Merck Millipore]) to analyze protein levels. Cells in 3D lrECM cultures were treated with ice-cold 5 mM EDTA in PBS and then lysed with RIPA buffer. Cell lysates were collected after sonication and centrifugation. Proteins were separated by molecular weight using SDS-polyacrylamide gel electrophoresis (PAGE) or Nu-PAGE Bis-Tris Gels (Thermo Fisher Scientific) and transferred to a PVDF membrane (Merck Millipore). The Membrane was blocked with Odyssey blocking buffer (LI-COR Biosciences) and then probed with a primary antibody at 4°C overnight. After washing with Tris-buffered saline containing Tween 20 (TBST; 25 mM Tris [pH 7.4], 120 mM NaCl, 3 mM KCl and 0.1% Tween 20), the membrane was incubated with an IRDye secondary antibody for 1 h at room temperature (RT), and then washed with TBST and PBS. The signals were detected with an Odyssey Imaging System (LI-COR Biosciences).

**Antibodies**

The following primary antibodies were used for immunoblotting: anti-Rab27a polyclonal antibody (17817-1-AP, Proteintech), anti-Rab27b polyclonal antibody (13412-1-AP, Proteintech), anti-β-actin monoclonal antibody (A5441, Sigma-Aldrich), anti-Xpress monoclonal antibody (R910-25, Thermo Fisher Scientific), anti-EREG monoclonal antibody (D4O51, Cell Signaling Technology) for western blotting, anti-EREG polyclonal antibody (PA5-24727, Thermo Fisher Scientific) for IHC staining, anti-EGFR monoclonal antibody (D38B1, Cell Signaling Technology), and anti-p-EGFR monoclonal antibody (Tyr1068; D7A5, Cell Signaling Technology).

**Immunofluorescence Staining**

Cells were fixed with 4% paraformaldehyde, permeabilized with 0.2% Triton X-100 in PBS for 10 min at RT, and then blocked with 5% BSA in PBS for 1 h at RT. Cells were incubated with a primary antibody at 4°C overnight, washed with PBS and incubated with an Alexa Fluor-conjugated secondary antibody (Thermo Fisher Scientific). F-actin was stained with phalloidin (Thermo Fisher Scientific). Cells were then washed with PBS and incubated with 0.5 µg/ml DAPI for 5 min to stain the nuclei.

Immunofluorescence staining of 3D lrECM-cultured cells was performed as described in a previous study.^1^ Briefly, cells from 3D lrECM cultures were plated on a glass slide (Matsunami), fixed with 4% paraformaldehyde and washed with 100 mM glycine in PBS. Cells were blocked with blocking buffer (10% goat serum, 1% goat anti-mouse lgG F[ab’]_2_ fragment [Thermo Fisher Scientific] in IF buffer [0.2% Triton X-100, 0.1% BSA, 0.05% Tween 20, 0.05% NaN_3_ in PBS]) for 1.5 h at RT in a humidified chamber and subsequently incubated with phalloidin. Cells were then washed with PBS, incubated with DAPI for 5 min, washed with PBS for 10 min, and mounted.

Fluorescence images were acquired using a Leica True Confocal Scanning (TCS) SP8 microscope system (Leica Microsystems).

**Apoptosis Assay**

An Annexin V-FITC Apoptosis Detection Kit (Abcam) was used to analyze apoptosis. Cells were harvested with trypsin-EDTA (Nacalai Tesque) and incubated with Annexin V-FITC for 10 min at RT in the dark. Cells not-subjected to Annexin V-FITC staining were prepared as a negative control. Fluorescence was analyzed using a FACSAria III flow cytometer (BD Biosciences). The final mean FITC intensity in each group was calculated as follows: (mean FITC intensity in the Annexin V-FITC-stained group) - (mean FITC intensity in the negative control group)

**SiRNAs and Transfection**

Lipofectamine RNAiMax 2.5 μl and 50 pmol siRNA in 100 μl Opti-MEM per 1 ml culture medium were mixed and incubated for 20 min at RT. Then, the mixture was added to 5 × 10^4^ ~ 2 × 10^5^ cells. Each assay was performed at 72 h after transfection.

***In Vivo* Experiment**

For the establishment of orthotopic tumors, 5-week-old female BALB/c-nu/nu nude mice were obtained from Hokudo. U87MG-Luc cells transfected with shCTL or shRab27b #1 were resuspended (5 × 10^5^ cells in 5 μl of PBS) and injected into the brains of nude mice using a Hamilton syringe (Hamilton Co.). Tumor cells were injected at a location 2 mm to the right and 1 mm anterior to the bregma at a depth of 3 mm below the surface of the skull. Cells were injected at a rate of 2.5 μl per minute. U87MG-Luc shCTL or shRab27b tumor-bearing mice were further divided into 2 groups (12-15 mice per group): a sham irradiated control group and a group treated with 4 Gy × 4 times of whole-brain fractionated irradiation on day 7 to 10 after the implantation of tumor cells. The numbers of mice in each group were shCTL, 9 mice; shRab27b, 6 mice; shCTL IR, 7 mice; shRab27b IR, 8 mice. The bioluminescence of the mice was measured using an *in vivo* imaging system (IVIS Spectrum CT; PerkinElmer) following an intraperitoneal injection of 50 mg/kg D-luciferin (Wako). Mice were monitored until signs of prostration (such as poor ambulation and the loss of over 20% of the body mass) were observed, and perfusion was performed with PBS. Kaplan-Meier survival curves were generated using GraphPad Prism.

**IHC Staining**

After blood perfusion with PBS following 4% paraformaldehyde, the mouse brains were obtained, fixed overnight at 4˚C, dehydrated through an ethanol gradient, and embedded in paraffin blocks for IHC. Four-micrometer sections from the paraffin blocks were mounted onto microscope slides. For antigen retrieval, the slides with mouse brain sections were incubated with antigen unmasking solution (Vector Laboratories) at 95 ˚C for 30 min. Endogenous peroxidase activity was quenched by incubation with 3% H_2_O_2_ for 10 min at RT. The slides were incubated with 2% blocking buffer (Roche) for 1 h and then incubated with a primary antibody for 2 h at RT. After three washes with 0.05% Tween 20 in PBS, the signals were amplified by Super Sensitive IHC Detection Systems (BioGenex). The sections were stained with the horseradish peroxidase secondary antibody. After two washes, the sections were counterstained with hematoxylin (Muto Pure Chemicals). The images were color deconvoluted to DAB images using Fiji (ImageJ), and then the optical densities (ODs) of Rab27b and EREG staining were quantified by the following formula: OD = log (max intensity/mean intensity), where max intensity is equal to 255 for 8-bit images. The relative OD was calculated as follows: (the OD of a DAB image with a primary antibody staining) – (the OD of a DAB image with negative staining).

**Coculture**

For 2D cocultures, H4 or U87MG cells were plated on cell culture inserts with a pore size of 0.4 μm (Millicell) and transfected with siRNA. After 24 h incubation, H4 cells were seeded in the lower chamber of 6-well plates, and the cell culture inserts (upper chamber) were placed to initiate coculture. After an incubation for 3 days, H4 cells were harvested and counted.

For 3D cocultures, H4 or U87MG cells were plated and transfected with siRNA in 24-well plates. After a 24-h incubation, these cells (lower part) were seeded on 3D lrECM and sandwich-coated with an additional lrECM layer. Then, H4-Luc cells (upper part) were seeded on the Matrigel and fed with 100 μl of medium containing 5% Matrigel. After 3 days of incubation, 100 μl of 10 mg/ml luciferin were added. After incubation for 1 h at 37°C, luminescence was measured using a CLARIOstar (BMG Labtech).

**References**

1. Lee GY, Kenny PA, Lee EH, Bissell MJ. Three-dimensional culture models of normal and malignant breast epithelial cells. *Nat Methods*. 2007;4(4):359-365.

**Supplementary Table 1. List of sequences**

**Supplementary Figure**

**Supplementary Figure S1**


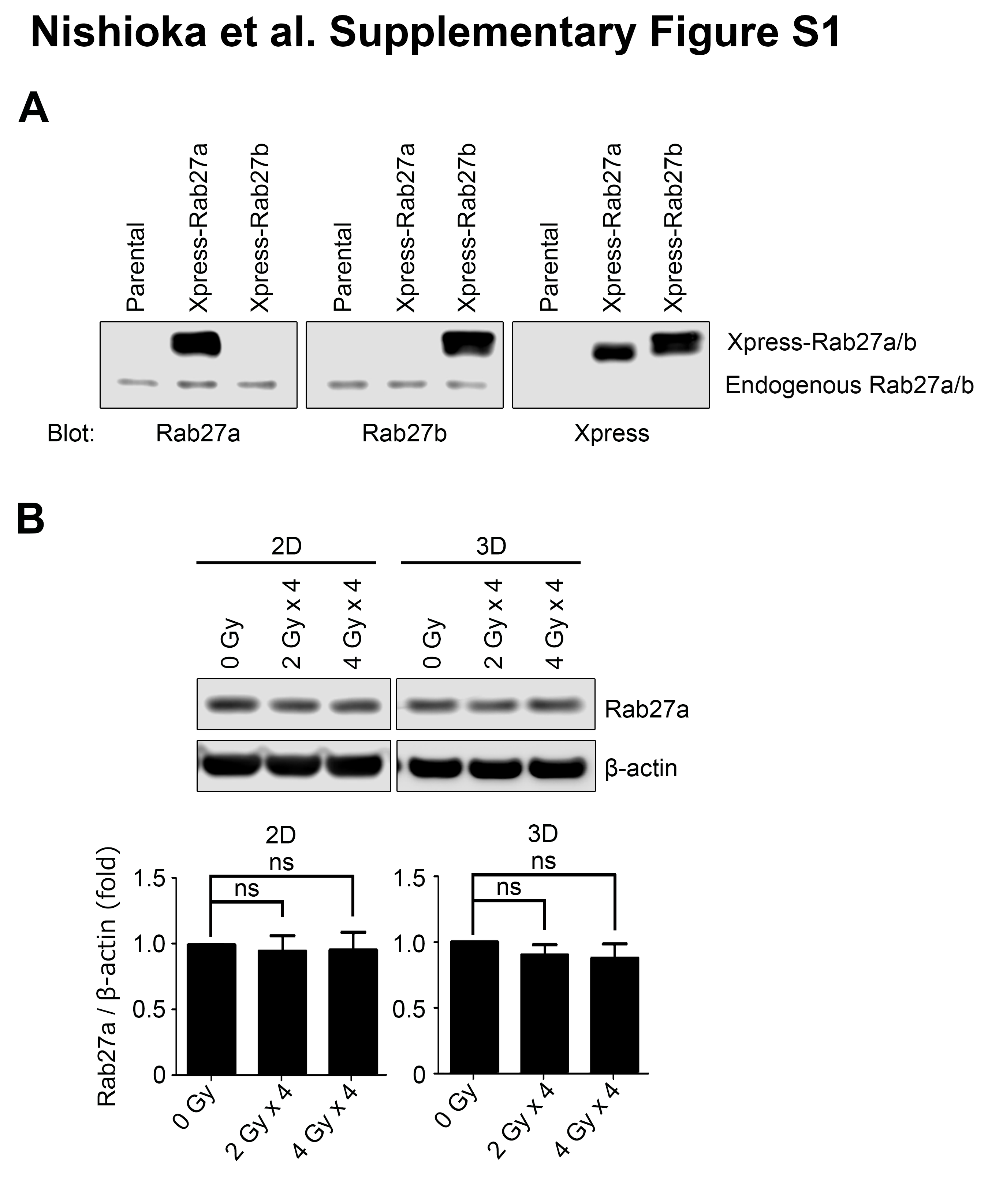


**Supplementary Figure S1.** Rab27a expression is not affected by IR treatment. **(A)** The specificity of antibodies for Rab27a and Rab27b was validated with lysates from parental U87MG cells overexpressing Xpress-Rab27a or Xpress-Rab27b by performing western blotting with the anti-Rab27a polyclonal antibody (Proteintech; 17817-1-AP), anti-Rab27b polyclonal antibody (Proteintech; 13412-1-AP), and Xpress monoclonal antibody. **(B)** The relative protein levels of Rab27a were measured in 2D or 3D lrECM cultures of U87MG cells after fractionated irradiation by western blotting. The intensities of the Rab27a band were normalized to β-actin. Columns, mean (n=3); bars, SE; ns, not significant.

**Supplementary Figure S2**


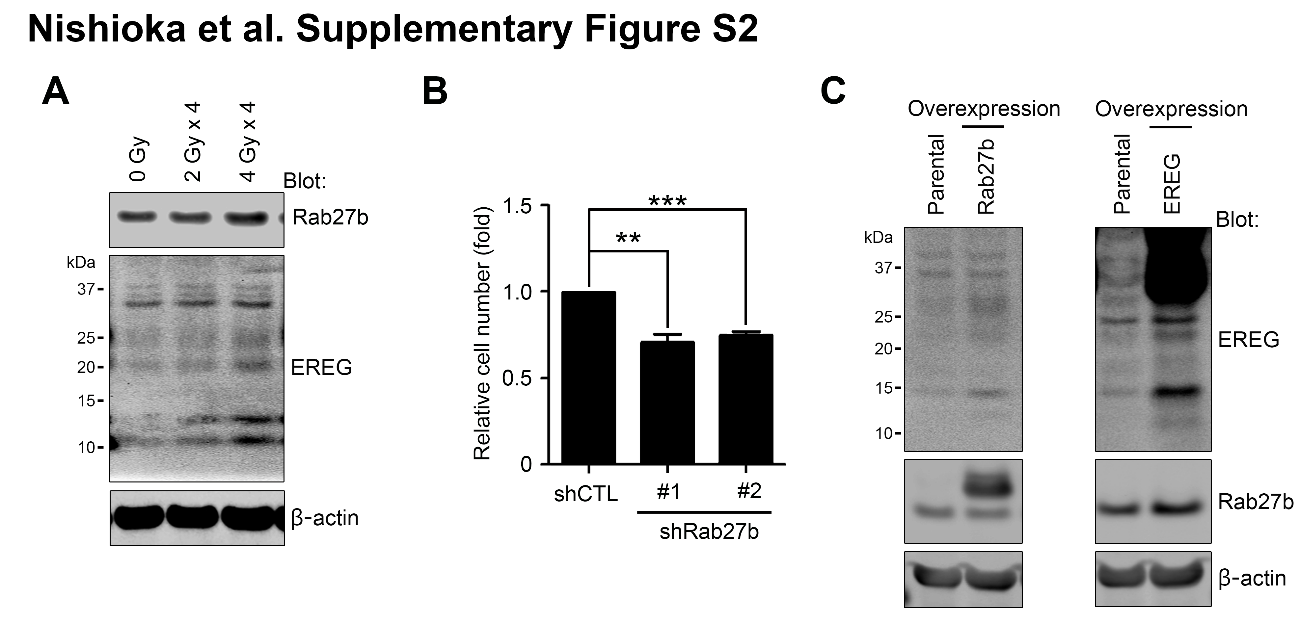


**Supplementary Figure S2.** Rab27b promotes radioresistance and co-regulated with EREG in U118MG cells **(A)** The levels of Rab27b and EREG proteins were measured after fractionated irradiation (0 Gy, 2 Gy × 4 or 4 Gy × 4) in 3D lrECM-cultured U118MG cells. **(B)** Relative cell numbers of control (shCTL) or Rab27b knockdown (shRab27b #1 or #2) exposed to IR were measured by cell counting and were normalized to the control group. Columns, mean (n=3). **(C)** Rab27b and EREG levels were measured in U118MG cells stably overexpressing Xpress-Rab27b or EREG-Venus using western blotting.

**Supplementary Figure S3**

**
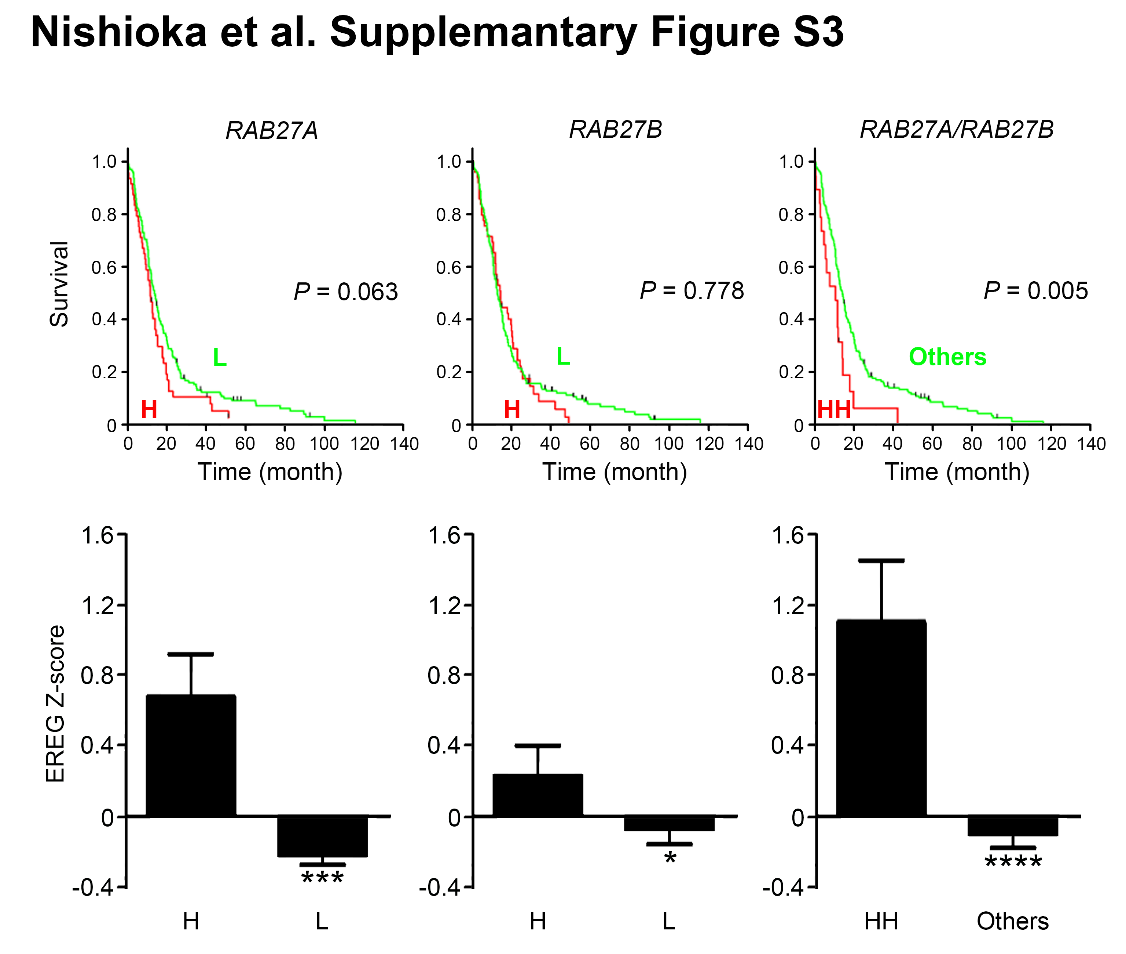
**

**Supplementary Figure S3.** TCGA analysis shows that Rab27 correlates with EREG expression and poor prognosis of patients with GBM. Kaplan-Meier survival curves (upper panels) were generated for patients stratified by the expression levels of *RAB27A* or *RAB27B* either alone or in combination using TCGA microarray data from 206 human patients with GBM. The average Z-scores for EREG in each group were also calculated (lower panels). H, high; L, low; HH, *RAB27A*/*RAB27B*-high/high. Columns, mean; bars, SE; **P*< 0.05, ****P* < 0.001, *****P* < 1 × 10^-10^, Brunner-Munzel test.

**Supplementary Figure S4**


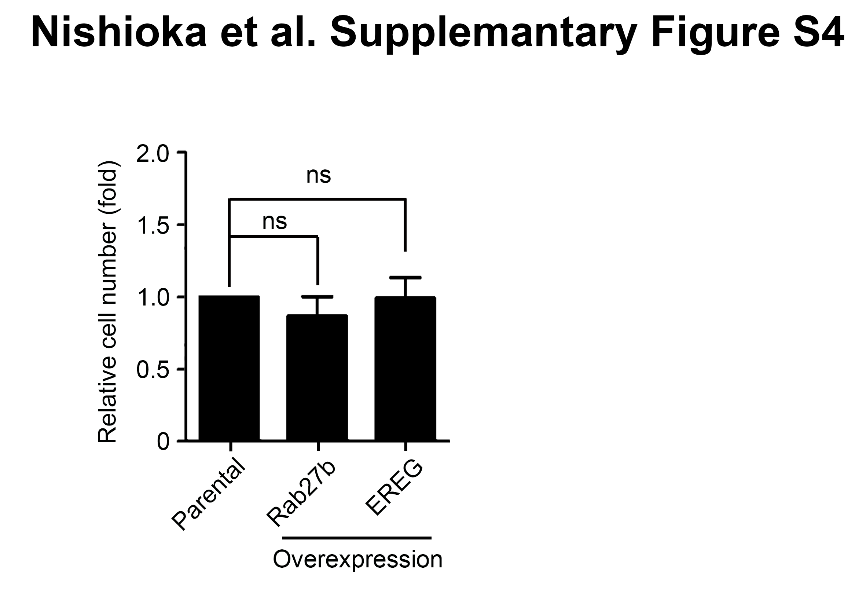


**Supplementary Figure S4.** Stable overexpression of Rab27b or EREG does not affect the radioresistance of U87MG cells. Xpress-Rab27b or EREG-Venus was stably expressed in U87MG cells. Cell viability after IR treatment was analyzed by cell counting. Columns, mean (n=3); bars, SE; ns, not significant.
